# FUT8 and Protein Core Fucosylation in Tumours: From Diagnosis to Treatment

**DOI:** 10.7150/jca.58268

**Published:** 2021-05-13

**Authors:** Chengcheng Liao, Jiaxing An, Suqin Yi, Zhangxue Tan, Hui Wang, Hao Li, Xiaoyan Guan, Jianguo Liu, Qian Wang

**Affiliations:** 1 Special Key Laboratory of Oral Disease Research, Higher Education Institution in Guizhou Province, School of Stomatology, Zunyi Medical University, Zunyi 563006, China.; 2 Microbial Resources and Drug Development Key Laboratory of Guizhou Tertiary Institution, Life Sciences Institute, Zunyi Medical University, Zunyi 563006, China.; 3 Department of Gastroenterology, Affiliated Hospital of Zunyi Medical University, Zunyi 563000, China.; 4 Department of Orthodontics II, Hospital of Stomatology, Zunyi Medical University, Zunyi 563000, China.

**Keywords:** FUT8, core fucosylation, EGFR, TGF-β receptor, E-cadherin, PD1/PD-L1, α3β1 integrin.

## Abstract

Glycosylation changes are key molecular events in tumorigenesis, progression and glycosyltransferases play a vital role in the this process. FUT8 belongs to the fucosyltransferase family and is the key enzyme involved in N-glycan core fucosylation. FUT8 and/or core fucosylated proteins are frequently upregulated in liver, lung, colorectal, pancreas, prostate,breast, oral cavity, oesophagus, and thyroid tumours, diffuse large B-cell lymphoma, ependymoma, medulloblastoma and glioblastoma multiforme and downregulated in gastric cancer. They can be used as markers of cancer diagnosis, occurrence, progression and prognosis. Core fucosylated EGFR, TGFBR, E-cadherin, PD1/PD-L1 and α3β1 integrin are potential targets for tumour therapy. In addition, IGg1 antibody defucosylation can improve antibody affinity, which is another aspect of FUT8 that could be applied to tumour therapy.

## 1. Introduction

Glycosylation is a ubiquitous modification that occurs on the proteins and lipids of all living cells, and these polysaccharides are essential for life. Protein glycosylation is involved in the basic molecular and cellular biological processes in the development of cancer, such as cell signalling and communication, tumour cell migration and invasion, cell matrix interaction, tumour angiogenesis, immune regulation and metastasis formation 1. To date, more than 180 glycosyltransferase genes have been discovered to be involved in the biosynthesis of glycans 2-3. It has become possible to manipulate glycosyltransferases to modify the structure of oligosaccharides to examine the effects of these modifications on certain events 4-5. Fucosyltransferase (FUT) is essential in many physiological and pathological activities, such as inflammation, bacterial and viral infections, tumour metastasis, and genetic diseases 6, and it is involved in regulating the fucosylation of O-glycans and N-glycans.

To date, 13 species of fucosyltransferase have been identified and they are divided into five categories: The first category, including FUT1 and FUT2, relates to the synthesis of α-1, 2 fucosidic bonds; the second category, including FUT3, 4, 5, 6, 7 and 9, is related to the synthesis of α-1, 3/4 fucoside bonds; the third category, mainly FUT8, is related to the synthesis of α-1, 6 fucoside bonds; and the fourth category includes FUT10 and FUT11. The enzyme activities of FUT10/11 are still under debate, but there is a paper showing the activity of these enzymes 7. The last category includes Pofut1 and Pofut2, which modify EGF-like domains and thrombospondin repeats (TSRs), respectively 8. Current research shows that FUT8, Pofut1 and Pofut2 are essential for the normal development of mice, indicating the importance of some members of FUTs in the normal physiological functions of the body 9. FUTs are involved in tumour regulation, especially FUT8, which is considered to be directly related to tumours 9-10.

The FUT8 gene is located on chromosome 14q24.3. Its chromosome location is different from any other fucosyltransferase genes reported so far, and its structure is also quite different, suggesting that FUT8 may have unique biological significance 11. No oligosaccharide structure with a core fucose was found in mice after FUT8 gene knockout, suggesting that FUT8 may be the only fucosyltransferase involved in core fucosylation 12. Mammalian FUT8 is a type II transmembrane glycoprotein, which is mainly concentrated in the Golgi body 13. It is a catalytic enzyme whose function is to transfer GDP fucose to the initial N-acetylglucosamine (GlcNAc) residue of the N-glycan core by forming α-1,6 glycosidic bonds, which constitutes the core fucose 14.

FUT8 consists of a catalytic domain, an N-terminal α-helical domain and a C-terminal Src homology 3 (SH3) domain. The SH3 domain is usually mediates protein-protein interactions by recognizing a proline-rich peptides in cytoplasmic signal transduction molecules 15. No other glycosyltransferases have been found to have SH3 domains. The SH3 domain binds to ribophorin 1 (RPN1) to strictly control the catalytic activity and positioning of FUT8, thereby promoting the activity of FUT8 and the core fucosylation 16. FUT8 follows the S_N_2 mechanism and unfolds a series of loops and an α-helix, which all contribute to the formation of binding sites, and an exosite composed of one loop structure and one SH3 domain is responsible for recognizing branched sugars 17. When bound to the acceptors, FUT8 requires the presence of a terminal GlcNAc moiety on the α1,3 arm of the N-glycan 18. The process of FUT8 capturing the substrate and the formation of the salt bridge between GDP and the two flow cycles are largely driven by Arg365 19. In addition, Glu273 and Lys369 of FUT8 directly play a catalytic role (Glu273 acts as a catalytic base, and Lys369 transfers a proton from Glu273 to the leaving phosphate group of the GDP-fuc substrate) 19.

In this review, we describe the diagnostic value of FUT8 and glycoproteins with core fucosylation for liver, lung, colorectal, pancreas, prostate, oral cavity, oesophagus, thyroid and stomach tumours (Table 1). More importantly, many pivotal glycoproteins on human tumour cells are highly core fucosylated, and core fucosylation is essential for the functions of these proteins. We summarized the existing evidence and discussed the impact of these core fucosylated glycoproteins on tumors to further clarify the feasibility of targeting FUT8 for the treatment of tumors. In addition, applying the FUT8 knockout mammalian cell lines to antibody production can obtain antitumour monoclonal antibodies with better performance, defucosylated IGg1 antibodies have been applied in tumour therapy.

## 2. FUT8 and glycoprotein core fucosylation as tumour diagnostic markers

FUT8 is frequently upregulated in human tumours 20-21. A recently published meta-analysis showed that FUT8 expression levels are associated with the overall survival of breast cancer, non-small-cell lung cancer, glioma, diffuse large B-cell lymphoma, and gastric cancer 21. FUT8 is also related to the disease-free survival of breast cancer, non-small cell lung cancer and colorectal cancer, and the recurrence-free survival of pancreatic ductal adenocarcinoma 22. Of course, the diagnostic value of FUT8 in tumours is not limited to its abnormal expression of itself, and the changes in glycoprotein core fucosylation mediated by FUT8 are also very valuable diagnostic indicators.

### 2.1. AFP-L3, FUT8 and core fucosylated haptoglobin, ceruloplasmin for hepatocellular carcinoma diagnosis

Hepatocellular carcinoma (HCC) is the most common type of liver cancer. Currently, only α-fetoprotein (AFP), core-focused AFP (AFP-L3) and des-gamma-carboxy prothrombin (DCP) are used as biomarkers for HCC 23. AFP-L3 has a high sensitivity for early-stage HCC 24: reflects the progression and migration of HCC, such as DCP and AFP 25; and can be used to predict the risk of HCC in nonalcoholic fatty liver disease (NAFLD) patients 26. More importantly, the increase in AFP-L3 can further rule out the possibility of other liver diseases, such as hepatitis and cirrhosis, thereby reducing the false-positive results of AFP in the diagnosis of HCC 27-28. AFP-L3 has been approved by the U.S. Food and Drug Administration (FDA) as a diagnostic serum biomarker for HCC.

FUT8 participates in AFP core fucosylation 29, and can be used as a marker to assess HCC progression 30. The expression of FUT8 promotes the proliferation and invasion of HCC 31, and mediates multidrug resistance in human liver cancer through the PI3K/Akt signalling pathway 32. FUT8 has good correlation with AFP. A recent study showed that in HCC with low AFP levels, several sialylated rather than core-fucosylated triantennary were glycans uniquely increase, while in high AFP HCC, many core-fucosylated biantennary, mixed glycans and dimeric glycans were uniquely increased 33.

Haptoglobin (Hp) is an abundant human plasma protein. The main biological function of Hp is to remove the haemoglobin (Hb) with high affinity, thereby preventing Hb-mediated kidney and blood vessel damage, iron loss, and the oxidation of heme-based proteins and membrane lipids. Hp also regulates angiogenesis, nitric oxide homeostasis, immune response and prostaglandin synthesis 34. An increased degree of Hp core fucosylation can distinguish patients with early HCC and those with liver cirrhosis and provide a potential marker for the early detection and prediction of HCC in patients with liver cirrhosis 35. Only a small amount of core fucosylation is detected in haptoglobin in chronic disease patients 36, making core fucosylated haptoglobin a valuable HCC indicator similar to AFP-L3.

The expression of ceruloplasmin in HCC is higher than that in liver cirrhosis, which is relevant to the diagnostic value of early detection of HCC, and can be used for prognostic evaluation of the survival rate 37. There are four core fucosylation sites (sites N138, 358, 397, and 762) in ceruloplasmin, and the core fucosylation rate of three sites in alcohol-related HCC was significantly higher than that in alcohol-related cirrhosis 38. In addition, the core fucosylation of α-1-antitrypsin (A1AT) is an HCC-specific glycoprotein that can predict HCC metastasis 39.

### 2.2. FUT8 and core fucosylated E-cadherin as biomarkers for lung cancer

FUT8 mRNA may serve as a potential plasma biomarker for lung cancer patients 40. The expression of FUT8 is elevated in non-small-cell lung cancer (NSCLC), and the expression of FUT8 is related to the tumour metastasis, tumour stage (N0), male sex, disease recurrence and a poor survival rate of NSCLC patients 21, 41-42. Mass spectrometry (MS) analysis of N-glycans compared cancer tissues with adjacent healthy tissues and found that advanced disease stages were mostly observed to have core fucosylated N-glycans with additional fucose (Fuc) residues 43. In addition, the core fucosylated E-cadherin can be used as a prognostic indicator for lung cancer patients 44, and an AlphaLISA analysis method for quantifying serum core-fucosylated E-cadherin as a biomarker of metastatic lung adenocarcinoma has been developed 45.

### 2.3. Diagnostic value of FUT8 and glycoprotein core fucosylation in colorectal cancer

The expression of E-cadherin and FUT8 are elevated in primary colorectal cancer (CRC) samples 46. In addition, the increased expression of FUT8 protein can be used as a prognostic marker for stage II and stage III CRC patients and is significantly associated with better disease-free survival (DFS) in p53-negative (but not positive) CRC tumours 47. And FUT8 expression was also related to microsatellite instability (MSI) 21. A total of 633 CRC patients and 478 age- and sex- matched controls were analyzed. In all stages of CRC (especially in stage 1), there are statistically significant differences in the plasma N-glycosylation, including the core fucosylated biantennary glycans F(6)A2G2 and F(6)A2G2S(6)1, which are significantly reduced 48. Changes in glycosylation significantly affect the structure and function of immunoglobulin G (IgG), and increased IgG N-glycan core-fucosylation, sialylation and sialo core-fucosylation were are possibly related to the development of CRC 49.

### 2.4. FUT8 and glycoprotein core fucosylation for the diagnosis of pancreatic cancer

Pancreatic ductal adenocarcinoma (PDAC) is the most common type of pancreatic cancer (PC). The expression of FUT8 in PDAC is significantly higher than that in normal pancreatic duct tissue, and is significantly related to lymph node metastasis and recurrence-free survival. In addition, FUT8 gene knockout can significantly reduce the invasion of PDAC cell lines and peritoneal metastasis 50. Human pancreatic ribonuclease 1 (RNase 1) is a glycoprotein mainly expressed in the pancreas. Although RNase 1 contains similar glycan structures in normal and tumour serum, there was a 40% increase in core fucosylation in the main sialylated biantennary glycans in the tumor serum RNase 1 51. Abnormal mucin overexpression is one of the characteristics of PC. A study found that FUT8 can stabilize the expression of MUC4 and MUC1 at the protein level through core fucosylation 52. This result suggests that core fucosylated MUC1/4 may also serve as a marker for the occurrence of PC. Chronic pancreatitis (CP) is a common risk factor for PDAC, and total serum core-Fuc-Hpt was shown to be an independent determinant of a subclinical CP diagnosis 53, which is meaningful to distinguish PDAC and CP patients.

### 2.5. FUT8 and core fucosylated PSA for the diagnosis of prostate cancer

Distinguishing between prostate cancer and benign prostatic hyperplasia (BPH) is a clinical problem, and finding and applying biomarkers is one of the solutions. Compared with BPH patients, the expression of core-fucosylated biantennary glycans was significantly increased in prostate cancer patients 54. Core fucosylated prostate-specific antigen (PSA) may be a diagnostic biomarker for differentiating prostate cancer from other prostate diseases, such as BPH 55, and both serum and urine tests for core fucosylated PSA are feasible options 56. In addition, the expression of FUT8 in aggressive tumours (Gleason 8 and above) is higher than that in non-invasive tumours (Gleason 6 and below) 57.

### 2.6. FUT8 and core fucosylated CD147 for the diagnosis of oral and oesophageal squamous cell carcinoma

The survival rates of oral squamous cell carcinoma (OSCC) and oesophageal squamous cell carcinoma (ESCC) are lower than 60% and 40%, respectively 58. A late-stage diagnosis and a lack of effective treatment strategies make OSCC and ESCC a serious health burden. Compared with normal oral epithelial tissue, the mRNA levels of FUT8 and core glycoprotein in OSCC patients are increased 59. The application of the glycoproteomics method discovered that core fucosylated CD147 is also considered a potential new tumour marker for the diagnosis of oral cancer 60. FUT8 expression in ESCC is higher than that in normal oesophageal tissue 61. FUT8 plays an anti-radiation-driven role in ESCC by core fucosylation of CD147, and it can be used as a marker to predict the radiotherapy response of ESCC patients 62.

### 2.7. FUT8 for the diagnosis of papillary thyroid carcinoma

In papillary thyroid carcinoma, high expression of FUT8 is related to an increased tumour volume and lymph node metastasis 63. The expression of FUT8 in normal follicles is very low, but FUT8 is highly expressed in 33.3% of papillary carcinomas, which is directly related to tumour size and lymph node metastasis. In contrast, this phenomenon is relatively rare in follicular carcinoma and anaplastic (undifferentiated) carcinoma, suggesting that the expression of FUT8 may be a key factor in the progression of papillary thyroid carcinoma (rather than follicular carcinoma), and a decrease in FUT8 expression may be related to anaplastic transformation 64.

### 2.8. FUT8 for the diagnosis of gastric cancer

FUT8 is considered to be related to gastric cancer (GC) 65. Interestingly, unlike other tumours, the expression of FUT8 is decreased in gastric cancer, and the upregulation of FUT8 expression can inhibit the proliferation of human gastric cancer cells 66. Researchers have shown that the level of core fucosylation residues of at least 9 N-glycan structures in GC is decreased significantly 67. More importantly, the high expression of FUT8 is related to a better overall survival rate of GC patients along with intestinal-type Lauren classification, male sex and TNM (stage I-III) 21.

### 2.9. FUT8 and glycoprotein core fucosylation for the diagnosis of breast cancer

FUT8 expression was found to be associated with a poor prognosis, advanced tumour stage and type of molecular classification (positive progesterone receptor and oestrogen receptor status), but was less associated with histological grade in breast cancer (BC) 21, 68, and two core fucosylated and agalactosylated glycans (m/z 1591, 1794) in IgG can clearly distinguish stage II patients from NCs 69. A core-fucosylated tetraantennary N-acetyllactosamine (F(6)A4G4 Lac1) is associated with poor clinical outcomes in BC, including lymph node metastasis, recurrent disease, and reduced survival 70.

### 2.10. FUT8 for the diagnosis of glioblastoma multiforme

Glioblastoma multiforme (GBM) is the most common and deadliest type of primary brain tumours. Although GBM does not generally form distant metastases, the spread of tumour cells to infiltrate the normal cerebral cortex can lead to death. Despite applying a combination of surgical resection, radiotherapy and chemotherapy, the median survival time is only 14 months 71. FUT8-AS1 is overexpressed in a subgroup of patients with glioblastoma multiforme (GBM) and is associated with even worse poor survival outcomes, information obtained by bioinformatics analysis of TCGA data 72. A meta-analysis study found that FUT8 expression was less related to pathological typing, but was associated with GBM patient age (≤40 years) and a shortened overall survival time of female patients 21.

### 2.11. FUT8 for the diagnosis of ependymoma

Ependymomas mainly involve the supratentorial brain, posterior fossa and spinal cord of the central nervous system, and can occur in both children and adults. At present, the treatment for patients with intracranial ependymoma is still surgical resection combined with radiotherapy, and the survival benefits of chemotherapy for ependymoma are still controversial. At present, there are no conventional molecular or tumour-specific immunohistochemical markers for ependymoma in clinical practice 73. The discovery of ependymoma diagnostic markers is of great significance for its clinical treatment. The current study found that high expression of FUT8 was related to ependymoma patient age (≤10 years) and tumour recurrence status 21. However, the diagnostic value of FUT8 in ependymomas remains to be discovered.

### 2.12. FUT8 for the diagnosis of medulloblastoma

Medulloblastoma (MB) originates in the brainstem or the part of the cerebellum located in the posterior fossa and is one of the most common malignant brain tumours in childhood 74. In MB, FUT8 expression levels were found to be associated with tumour stage, metastasis status, molecular classification (WNT and SHH molecular subgroups) and male sex 21.

### 2.13. FUT8 and glycoprotein core fucosylation for the diagnosis of the other cancers

The core fucosylation of copper transporter 1 (CTR1) plays an important role in the uptake of cisplatin (cDDP). The level of CTR1 core fucosylation in the serum of cDDP-resistant epithelial ovarian cancer (EOC) patients is significantly increased 75, which has guiding significance for the identification of patients with cDDP resistance. The upregulation of FUT8 and the downregulation of FUT1 and FUT2 were identified as the characteristics of metastatic melanoma 76. Glycan analysis of total serum reveals increased levels of core fucosylation in cholangiocarcinoma (CCA) 77.

## 3. FUT8-mediated EGFR core fucosylation for treatment

Epidermal growth factor receptor (EGFR) is also known as HER1 or ErbB1 and belongs to the ErbB family of tyrosine kinase receptors along with HER2/neu (ErbB2), HER3 (ErbB3) and HER4 (ErbB4) 78. EGFR can extensively affect the following downstream signalling pathways: RAS/RAF/MEK/MAPK/ERK, phosphatidylinositol 3-kinase (PI3K) /Akt, protein kinase C (PKC), Src and JAK/STAT pathways 79. These signalling cascades can affect cell gene expression, proliferation, angiogenesis, apoptosis inhibition, cell movement, metastasis, adhesion and angiogenesis 80. Dysregulation of EGFR expression and signalling pathways may play a key role in lung cancer, head and neck squamous cell carcinoma, and colon, pancreas, brain and breast cancer 81, and targeting for EGFR would be a great help to the treatment of these tumours. FUT8 gene knockout in mice resulted in early death, severe growth retardation, and emphysema-like changes in the lung, revealing that FUT8 is essential for the activation of EGFR 82-83.

The EGFR contains 20% carbohydrates calculated by molecular weight, N-glycosylation affects the structure and stability of this receptor, and the stability and balance of EGFR glycosylation is conducive to cross-cell signal transduction 84-85. In addition, the N-glycosylation of the extracellular domain of EGFR plays a key role in the binding of growth factors, monoclonal antibodies and EGFR dimers to the extracellular domain of mononuclear EGFR 86. N-glycosylation is the determinant of EGFR conformation in the cell membrane 87. These characteristics suggest that targeting EGFR N-glycosylation could be an effective therapy. The expression of FUT8 is related to EGF-mediated activation of JNK or ERK, and the core fucosylation of N-glycans is necessary for the binding of EGF to the receptor 88. Modified EGFR core fucosylation increases EGF-mediated cell growth and sensitivity to gefitinib 89. In contrast to FUT4 and FUT6, FUT8 can promote EGFR dimerization and phosphorylation in lung cancer cells 90. FUT8 promotes the aggressiveness and malignant TME of non-small cell lung cancer (NSCLC) through EGFR core fucosylation, which accelerates the proliferation of NSCLC cells 91. Drugs that can reduce EGFR core fucosylation are being used to treat tumours. 2-Fluoro-L-fucose (2FF) treatment reduces the fucosylation level of core membrane glycoproteins, thereby inhibiting downstream signals and inhibiting the progression of liver cancer 92.

In castration-resistant prostate cancer (CRPC), the expression of EGFR and FUT8 has a coordinated regulatory mechanism, and increased expression of FUT8 (because of resistance to androgen deprivation) increases the expression of EGFR; FUT8 plays a key role in converting nuclear receptor signal transduction (androgen receptor) into a cell surface receptor (EGFR) mechanism in prostate cancer cells evading androgen-induced cell death, which leads to an increased survival rate of prostate cancer cells under hormone depletion conditions 93. FUT8 silencing reduces the growth rate of TGP49 mouse pancreatic acinar cell carcinoma by attenuating EGFR phosphorylation and the EGFR-trypsin-PAR-2 pathway 94. FUT8 (-/-)) mice were treated with diethylnitrosamine (DEN) and pentobarbital (PB). In FUT8 (+/+) and FUT8 (+/-) mice, after DEN and PB treatment, the expression of FUT8 was increased, which induced multiple large, vascularized nodules. However, the formation of HCC in FUT8 (-/-) mice was almost completely inhibited. The inhibitory effect of FUT8 deficiency on tumorigenesis was also confirmed by the disappearance of tumour formation by human hepatoma cell line cells lacking FUT8 in xenograft tumour models. In addition, the deletion of the FUT8 gene leads to a weakened response of the HepG2 cell line to epidermal growth factor (EGF) and hepatocyte growth factor (HGF), which provides a possible mechanism by which FUT8 participates in liver cancer 95. In summary, the core glycosylation mediated by FUT8 has a significant impact on EGFR signalling and is a therapeutic target for cancer.

## 4. FUT8-mediated TGFBR core fucosylation for treatment

TGF-β1, 2 and 3 are receptor ligands with similar biological activity, and are important in regulating proliferation, migration, differentiation, and apoptosis. There are also three TGF-β receptors named TGFBR1, 2, and 3. Canonical TGF-β signalling occurs when TGF-β1, 2 or 3 binds to TGFBR2, and then TGFBR1 is recruited and phosphorylated. After that, phosphorylated TGFBR1 increases SMAD2/3 phosphorylation, and then activated SMAD2/3 can form a complex with a common SMAD mediator, i.e., SMAD4, and translocate into the nucleus where the heteromeric complex modulates the transcription of target genes, which then recruit SMAD4 and translocate to the nucleus where it regulates the transcription of TGF-β target genes 96-98. Both receptors are N-glycoproteins with potential N-linked glycosites at Asn-70, 94, and 154 of TGFBR2 and at Asn-45 of TGFBR1, and TGF-β sensitivity can be regulated by N-linked glycosylation of TGFBR 99-100.

A recent study showed that two conserved asparagine residues, Asn-70 and 94, are N-glycosylated and are essential for the cell-surface transport of TGFBR2 99. First, core fucosylation may cause conformational alterations of TGF-β receptors. In addition, TGFBR N-glycosylation is necessary for its transport on the cell surface 101. In the TGF-β/Smad2/3 signalling pathway, silencing FUT8 did not inhibit the protein expression of the TGF-β receptor and Smad2/3 protein; but it did significantly inhibited the phosphorylation of Smad2/3 and epithelial-mesenchymal transition (EMT). Similarly, in PDGF/ERK signal transduction, silencing FUT8 did not affect the expression of platelet-derived growth factor (PDGF) receptor and ERK protein, but it did significantly inhibit ERK phosphorylation and EMT 102. More importantly, even if the expression level of TGFBR increases, blocking core fucosylation can also weaken EMT 103. In addition, FUT8 is highly upregulated during the process of EMT induced by TGF-β, FUT8 overexpression stimulates EMT, and silencing FUT8 inhibits tumour invasion and migration 104. The core fucosylation of N-glycan in TGFBR is necessary for receptor function 105. Preventing FUT8-mediated TGFBR core fucosylation is of great significance for tumour EMT suppression, which is an important target for interference with EMT. Intervening in TGFBR core fucosylation is a feasible solution for inhibiting tumour EMT.

## 5. FUT8-mediated E-cadherin core fucosylation for treatment

E-cadherin belongs to the type-I cadherin family and is generally considered to be the prototype of all cadherins 106. Decreased expression of E-cadherin is a recurring finding in cancer, leading to tumour cell proliferation, survival, invasion and loss of epithelial cell polarity 107. The polysaccharide structure of E-cadherin has an important influence on its function. E-cadherin O-glycosylation in the cytoplasm blocks E-cadherin transport during the process of apoptosis, leading to decreased intercellular adhesion and endoplasmic reticulum stress 108. The N-glycosylation site of E-cadherin located in extracellular region 4 (EC4) changes the molecular structure of E-cadherin, thereby destabilizing adherens junctions 109. In addition, there are two N-glycosylation sites on E-cadherin located in EC5 109. The N-glycan on Asn 633 is essential for the folding, transport and correct expression of E-cadherin 110. The degree of E-cadherin N-glycan branching degree is inversely related to the establishment of cell adhesion 111. In fact, E-cadherin N-glycosylation is more common than the other two known E-cadherin posttranslational modifications 107. N-acetylglucosamine transferase III (GnT-III), N-acetylglucosamine transferase V (GnT-V) and FUT8 play important roles in N-glycan remodelling of E-cadherin 112. Therefore, FUT8-mediated E-cadherin core fucosylation may have an important impact on tumor cell the malignant phenotype of tumour cells.

FUT8 plays an important role in the E-cadherin redistribution and downstream signal regulation. Core fucosylation of E-cadherin could significantly hinder the three-dimensional conformation effect of N-glycan on E-cadherin, resulting in conformational asymmetry, thereby inhibiting the function of E-cadherin 43. In colon cancer WiDr cells transfected with FUT8, FUT8 participates in the emergence of a low-molecular-weight E-cadherin population, regulates the total amount of Ecadherin, regulates the transfer potential and the stability of cell-to-cell contact 46. The lack of FUT8 inhibits the FAK/integrin pathway by inhibiting E-cadherin core fucosylation, reducing the accumulation of nuclear chain proteins and inhibiting the expression of MMP-2 and MMP-9, which inhibits the adhesion, migration and invasion of breast cancer cells 112. In addition, activation of the Wnt/β-catenin signalling pathway upregulates the expression level of FUT8, thereby inducing an increase in stemness and EMT in breast cancer cells 113. In human low and highly metastatic giant lung cancer lines (95C, 95D), core-fucosylated E-cadherin regulates nuclear β-catenin accumulation 114, and Src activation and induces epithelial-mesenchymal transition (EMT)-like processes 115. Deoxycholic acid impairs glycosylation and core fucosylation processes in oesophageal epithelial cells, reducing the expression of E-cadherin 116. Therefore, intervention into FUT8-mediated E-cadherin core fucosylation in tumours is of great significance for E-cadherin loss and relocation.

## 6. FUT8-mediated PD-1/PD-L1 core fucosylation for treatment

Tumour cells can evade T-cell-mediated cytotoxicity by inhibiting programmed cell death protein 1 (PD-1)/programmed cell death ligand 1 (PD-L1) immune checkpoints 117. Clinical trials have shown that anti-PD-1/PD-L1 monoclonal antibodies are effective in treating melanoma, non-small-cell lung cancer (NSCLC), renal cell carcinoma (RCC), and bladder cancer 118. In fact, both PD-1 (NM-005018) and PD-L1 (NM-001267706) are glycoproteins with four core-fucosylated N-glycans, namely, N4, 49, 58, 116 and N35, 192, 200, 219, respectively 119-120. Glycosylation of PD-1, especially at the N58 site, plays a crucial role in mediating its interaction with PD-L1 121. PD-L1 N-glycosylation sites at N192, N200 and N219 antagonize GSK3β binding and decrease the phosphorylation-dependent proteasomal degradation of PD-L1 by β-TrCP 122. Inhibition of the core transferase FUT8 can reduce the expression of PD-1 on the cell surface, enhance the activation of T cells, and thus more effectively eradicate the tumours 123. The absence of FUT8 significantly enhanced the ubiquitination of PD-1, leading to the degradation of PD-1 in the proteasome 124. Therefore, interference into PD-1/PD-L1 core fucosylation mediated by FUT8 is valuable for blocking PD-1/PD-L1-mediated tumour immune escape.

## 7. Core fucosylated α3β1 integrin for treatment

Integrins belong to the family of cell-adhesion molecules (CAMs), which are include two noncovalently linked heterodimer α and β subunits, mediate cell-to-cell and cell-to-extracellular matrix (ECM) adhesion, and provide adhesion and traction during cell movement 125-126. In recent years, abnormal expression of integrin has been found in many tumours, and it has an impact on the differentiation, migration, proliferation and angiogenesis of tumour cells. In addition, research on integrin-targeted drugs is of great significance for the clinical treatment of tumours 127. The activity of integrins is strongly influenced by glycosylation events and glycan-mediated interactions 128. Integrins contain more than 20 potential glycosylation sites 129. The existence of an N-glycan structure is essential for integrin heterodimerization, conformational stability, expression on the cell membrane, and interaction with ligands 128. N-glycosylation is dynamic. The remodelling of N-glycosyls due to the action of glycosyltransferase can regulate the binding of integrins to substrates and play a key role in cell adhesion and migration 130. FUT8 is essential for α3β1 integrin core fucosylation. FUT8-mediated α3β1 integrin core fucosylation can induce laminin 5-induced migration and intracellular signal transduction 131. N-glycan core fucosylation on the cell surface acceptor can promote the self-assembly of cancer cells mediated by cyclo-RGDfK (TPP) to form three-dimensional multicellular tumour spheroids 132.

## 8. Defucosylated IGg1 improves treatment performance

Monoclonal antibody therapy is increasingly used to treat cancer, and market data predict that the growth rate of monoclonal antibodies will be higher than any other therapeutic biological drug 133. Most therapeutic antibodies are of the human IgG1 subtype. Complement-dependent cytotoxicity and antibody-dependent cytotoxicity (ADCC) are the main mechanisms by which antibodies activate the immune system 134. Natural killer cells activate ADCC mainly by binding to the FcγRIIIa receptor via the FC part of the antibody 135. The effect of natural killer (NK) cell recruitment and activation is determined by the affinity of IgG to FcγRIIIa, which is related to IGg FC oligosaccharide core fucosylation 136-137. Core fucosylation at asparagine 297 decreased ADCC activities by weakening its binding affinity to FcγRIIIa 138.

Mogamulizumab (POTELIGEO^®^) was the first glycoengineered antibody to reach the market; it is a defucosylated, humanized, anti‐chemokine receptor 4 mAb and is considered a milestone for therapeutic antibodies 139. Obinutuzumab (GA101 or Gazyva^®^) is the first glycoengineered therapeutic anti-CD20 antibody approved by the FDA in 2013 for the combined treatment of chronic lymphocytic leukaemia (CLL) and follicular lymphoma patients 140. A phase 1/2 GAUGUIN study showed that obinutuzumab monotherapy is effective in CLL and non-Hodgkin lymphoma patients with relapse/refractoriness 141-142. In phase 3 clinical trials, GA101 combined with chemotherapy can significantly prolonged overall survival and progression-free survival and improved complete remission rates in CLL patients 143.

Chinese hamster ovary (CHO) cells are used to produce 35.5% of all launched therapeutic antibodies 144, but the IgG1 antibody produced by CHO is extensively core fucosylated 145. After disrupting the FUT8 allele in the CHO/DG44 cell line, produced anti-CD20 IgG1 strongly binds to human FcRIIIa and dramatically enhances ADCC to approximately 100-fold higher than that of rituxan 146. In addition, the growth of FUT8 knockout CHO-S (FUT8 -/-) cells is similar to that of wild-type CHO-S cells 147 and antibodies produced by the FUT8 (-/-) CHO cell line without adverse phenotypic effects 145.

## 9. Conclusion and prospect

FUT8 and core fucosylated glycoproteins are frequently upregulated in human tumours and may be used as markers for tumour diagnosis (Table 1). In contrast, the expression of FUT8 is downregulated in gastric cancer, and the upregulation of FUT8 is associated with a better overall survival rate of GC patients 21. The functional implications of low FUT8 in gastric cancer have not been well studied. The core fucosylation changes of the N-glycan structure of some important receptors in gastric cancer may regulate signal transduction 66. In recent decades, new technologies such as MALDI mass spectrometry glycan imaging have been used to identify specific glycan changes in cancer tissues 148. Core fucosylated N-glycan structures have great potential to serve as cancer diagnosis, prognosis, and treatment monitoring biomarkers.

The significance of FUT8 expression changes in human tumours is still not fully elucidated, even though the existing studies have proved that the upregulation of FUT8 is prevalent in most human tumors. New fucosylation inhibitors (SGN-2FF, A2FF1P, B2FF1P, P-D-Rha6F2-1P and P-D-Rha6F3-1P) have been developed 20, but no evidence indicates that they are specific inhibitors that inhibit the synthesis of α-1,6 fucoside bonds. In some tumours, such as HNSCC and lung cancer, inhibiting the synthesis of α-1,3/4 fucoside bonds may promote tumour progression by improving EGFR activity 149-150. However, it should be noted that the inhibition of FUT8 may considerably impact normal human cells, thus limiting the application of FUT8 inhibitors in tumour therapy 149,151. Therefore, targeted therapy of key core fucosylated glycoproteins, such as the core fucosylation sites of EGFR, TGFBR, E-cadherin, PD-1/PDL-1 and α3β1 integrin, may be a more feasible approach.

## Figures and Tables

**Table 1:** FUT8 and core fucosylated glycoproteins as tumours diagnostic markers.

Tumours	Diagnostic markers	Application	Compared with normal level	Reference
Hepatocellular carcinoma (HCC)	AFP-L3	Diagnose HCC	↑	24-28
	FUT8	A marker to assess HCC progression	↑	30, 33
	Core fucosylated haptoglobin	Diagnose HCC	↑	34-36
	Core fucosylated ceruloplasmin	Distinguish between alcohol-related HCC and alcohol-related cirrhosis	↑	37, 38
	Core fucosylated α-1-antitrypsin (A1AT)	Predict HCC metastasis	↑	39
Pancreatic cancer (PC)	FUT8	Significantly related to lymph node metastasis and recurrence-free survival of PDAC patients	↑	21, 50
	Ribonuclease 1 (RNase 1)	Serum markers of pancreatic cancer	↑	53
	Total serum core-Fuc-Hpt	CP diagnosis	↑	53
Breast cancer	IgG m/z 1591, 1794	Distinguish stage II patients from NC	↑	69
	F(6)A4G4 Lac1	Poor clinical outcomes in breast cancer, including lymph node metastasis, recurrent disease, and reduced survival	↑	70
	FUT8	Associated with poor prognosis, disease-free survival, tumour stage and molecular classification	↑	21, 68
Lung cancer	FUT8 and FUT8 mRNA	Related to the poor survival rate, tumour stage (N0), male sex, and disease-free survival of NSCLC patients Potential plasma biomarker for lung cancer patients	↑	21, 40-42
	Core-fucosylated E-cadherin	Prognostic indicator for lung cancer patients	↑	44
	Core fucosylated N-glycans with additional fucose (Fuc) residue/s	Quickly distinguish between cancer and healthy lung tissue	↑	43
Colorectal cancer (CRC)	FUT8	As a prognostic marker for stage II and stage III CRC patients Related to disease-free survival and microsatellite instability	↑	21, 46, 47
	Core fucosylated bi-antennary glycans F(6)A2G2 and F(6)A2G2S(6)1	The risk of CRC	↑	48
	IgG Core-fucosylation and sialo Core-fucosylation	Possibly related to the development of CRC	↑	49
Oral squamous cell carcinoma (OSCC)	Core fucosylated CD147	Potential marker for the diagnosis of OSCC	↑	60
Epithelial ovarian cancer (EOC)	Core fucosylated CTR1	Guiding significance for the identification of patients with cDDP resistance	↑	71
Gastric cancer (GC)	FUT8	Associated with overall survival rate of GC patients in intestinal-type Lauren classification, male sex and TNM (stage I-III)	↓	21, 65, 66
Melanoma	FUT8	Diagnose melanoma metastasis	↑	72
Oesophageal squamous cell carcinoma (ESCC)	FUT8	A marker to predict the radiotherapy response of ESCC patients	↑	61, 62
	Core fucosylated CD147			
Prostate cancer	Core fucosylated prostate-specific antigen (PSA)	Diagnostic biomarker for differentiating prostate cancer from other prostate diseases	↑	56
	FUT8	Prostate cancer aggressiveness	↑	57
Papillary thyroid carcinoma	FUT8	Differential diagnosis from follicular carcinoma Related to tumour size and lymph node metastasis	↑	63, 64
Glioblastoma multiforme	FUT8-AS1	Associated with poor survival outcomes	↑	21,71
Ependymomas	FUT8	Related to ependymoma patient age (≤10 years) and tumour recurrence status	↑	21
Medulloblastoma	FUT8	Associated with tumour stage, metastasis status, molecular classification and male sex	↑	21
Diffuse large B-cell lymphoma	FUT8	Associated with overall survival	↑	21

Note: ↑, Increased compared to normal levels; ↓, decreased compared to normal levels.
